# Smartphone Microscopy of Parasite Eggs Accumulated into a Single Field of View

**DOI:** 10.4269/ajtmh.15-0427

**Published:** 2016-01-06

**Authors:** Stephen J. Sowerby, John A. Crump, Maree C. Johnstone, Kurt L. Krause, Philip C. Hill

**Affiliations:** Department of Biochemistry, University of Otago, Dunedin, New Zealand; Centre for Bioengineering and Nanomedicine, University of Otago, Dunedin, New Zealand; Centre for International Health, University of Otago, Dunedin, New Zealand; Webster Centre for Infectious Diseases, University of Otago, Dunedin, New Zealand

## Abstract

A Nokia Lumia 1020 cellular phone (Microsoft Corp., Auckland, New Zealand) was configured to image the ova of *Ascaris lumbricoides* converged into a single field of view but on different focal planes. The phone was programmed to acquire images at different distances and, using public domain computer software, composite images were created that brought all the eggs into sharp focus. This proof of concept informs a framework for field-deployable, point of care monitoring of soil-transmitted helminths.

## Introduction

Soil-transmitted helminths (STH) are nematode parasites that infect up to 2 billion people, with children in the developing world the most at risk from anemia, malnutrition, and developmental delay.^1^–^3^ Increasingly, STH are controlled by preventive chemotherapy through mass drug administration (MDA) within a paradigm of mapping disease distribution, monitoring MDA efficacy, strategic cessation of drug intervention, and postelimination surveillance.^4^,^5^

The monitoring of STH in resource-limited settings is constrained by poor laboratory infrastructure and capability leading to misdiagnosis.^6^ Parasite assessment has traditionally been done using the Kato–Katz method,^7^ the diagnostic standard of the World Health Organization,^8^ in which a fixed amount of stool (approximately 40 mg) is placed on a microscope slide and spread thinly over an area up to 25 mm in diameter. However, accurate diagnosis requires skilled microscopists, who can identify nematode ova in real time, across multiple fields of view (FOV).^9^,^10^ The optical resolution to identify nematode eggs (20–200 μm) requires magnifications of 40 to 100×,^9^,^10^ which constrain FOV to 4.45 and 1.78 mm in diameter and represents 31 and 197 nonoverlapping FOV on the Kato–Katz slide, respectively. The McMaster method is a flotation-based veterinary parasite diagnostic^10^–^12^ recently adapted for human application.^13^,^14^ Stool is homogenized in dense fluids causing the eggs to float to a common focal plane for microscopy. Gridlines that correspond to total searchable areas of 100 and 324 mm^2^ facilitate examination of 0.15 and 0.5 mL of captured fluid, respectively.^11^,^15^ The Flotation translation Cringoli (FLOTAC) apparatus also facilitates the microscopy of eggs over multiple grid-lined FOV after centrifuge-enhanced flotation.^16^

Manually finding and discriminating eggs from debris in stool by traditional microscopy is laborious and, combined with the rapid clearing of hookworm ova in the Kato–Katz method, limits quality-assured fecal egg counting and the monitoring of STH.^17^ Here, we provide proof of concept for an alternative approach that uses fluidic geometries to concentrate eggs into a single FOV^18^–^20^ and combines this with mobile phone digital photomicroscopy.^21^–^23^

## Methods

**Figure 1.**
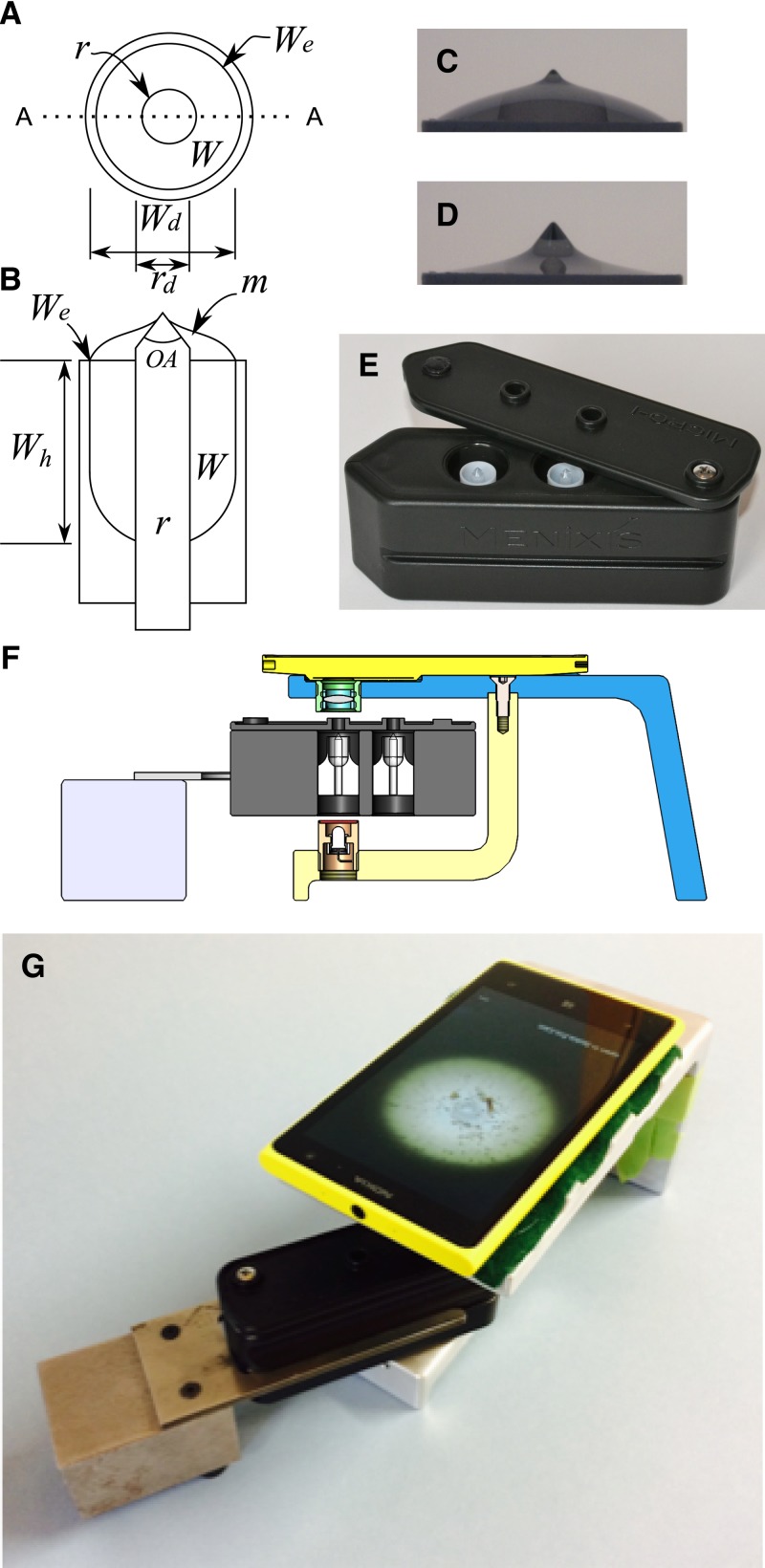
Axisymmetric meniscus (AxM) fluidics and Nokia Lumia 1020 Smartphone microscope. Schematic of the particle-accumulating fluid cell, (**A**) top view showing: rod (*r*); well (*W*); and well edge (*W*_e_) and (**B**) transverse cross section of (**A**) along the line A-A showing: the meniscus (*m*); rod diameter (*r*_d_ = 3 mm); well diameter (*W*_d_ = 8 mm); well depth (*W*_h_ = 12.1 mm). Profile images showing menisci in the apparatus shown in (**A** and **B**) showing the cone positioned with its apex (75° open angle (OA) 1.80 mm above the well edge and containing (**C**) 0.500 mL and (**D**) 0.480 mL of fluid. (**E**) Photograph of the cassette (30 × 35 × 100 mm) incorporating two fluid wells. (**F**) Schematic cross section showing the smartphone, cradle, cassette and cassette rack. (**G**) Photograph of the apparatus schematically shown in (**F**).

We generated a rotationally symmetrical (axisymmetric) meniscus (axisymmetric meniscus [AxM], Figure 1Andash;D

) to passively accumulate buoyant nematode ova into a single FOV.^18^–^20^ These menisci form about a tapered glass rod (3 mm diameter) located within a fluid well. The fluidic geometry is dominated by the surface tension at the liquid–air interface according to well-known physical laws of capillarity.^18^,^24^ Buoyant particles in the fluid wells accumulate within a fluid wedge formed between the AxM and the rod's tapered surface. Images of the rod magnified to occupy a single microscopic FOV and projected onto a 24″ screen permit up to 145× digital magnification.^18^ However, the vertical height of an AxM (∼1.8 mm, Figure 1C and D) is many times greater than the focal depth of the optics traditionally used to resolve helminth ova (e.g., a typical 10× objective lens = 11.2 μm)^25^ and variability in the fluid volume (Figure 1C and D)^18^,^24^ and interfering fecal debris can distribute particles at different focal depths. The problem of imaging three dimensionally is mitigated using images at different focal depths and fusing them into a single focused image using software.^26^,^27^

The fluid wells (Figure 1A–D) installed in *Micro-i* cassettes (Figure 1D, Techion Group, Dunedin, New Zealand), allow the analysis of 0.5 mL aliquots of processed stool, and have been previously characterized.^18^ We built a cassette rack and a bespoke cradle to hold a Nokia Lumia 1020 smartphone (Figure 1F and G). We mounted a double-convex objective lens (12 mm diameter, effective focal length 18 mm and a back focal length 16.81, center thickness 3.48 ± 0.01 mm, Edmund Optics, Singapore) 10.25 ± 0.02 mm in front of the camera. The cassette rack positioned the tip of the rod 14.90 ± 0.2 mm in front of the lens. A white light–emitting diode installed in the foot of the cradle-facilitated transillumination of the rod. A software application was created, which incorporated a graphical user interface, to preset the smartphone's digital zoom (1–5×) and specify the number and focal positions of images along the optical axis, which had an arbitrary range of 0–1,000, related to the physical limits of the smartphone's optical stage. To calibrate the microscope at the desired precapture digital zoom, the software was used to set the smartphone's optical stage to its midpoint (500) and the height of the cassette rack was adjusted so that the midsection of the image was in focus.

Before examining clinical specimens, the smartphone microscope was evaluated using a grid distortion target with 500 μm spacings (Thorlabs, Newton, NJ), a 1951 U.S. Air Force (USAF) resolution target and a depth-of-field target (Edmund Optics, Singapore). Images acquired using the smartphone microscope were ported to the public domain program ImageJ^28^ and combined into single images using the Extended Depth of Field plugin.^26^,^27^ A stool sample (2 g) from a person known to be positive for *A. lumbricoides*, was homogenized in saturated sodium chloride (50 mL) and strained through a 250 μm aperture sieve,^14^ which rendered the eggs buoyant in a fecal slurry. Aliquots of the mixed slurry were removed by pipette, applied to the fluid wells of the cassette and left to equilibrate for 10 minutes before imaging. The Human Ethics Committee of the University of Otago granted approval (code H13/017) for the use of anonymized stool specimens, which would have otherwise been discarded. The sample used here was sourced and assessed by a competent technician from an independent laboratory (Auckland DHB LabPlus, Auckland, New Zealand).

## Results

**Figure 2.**
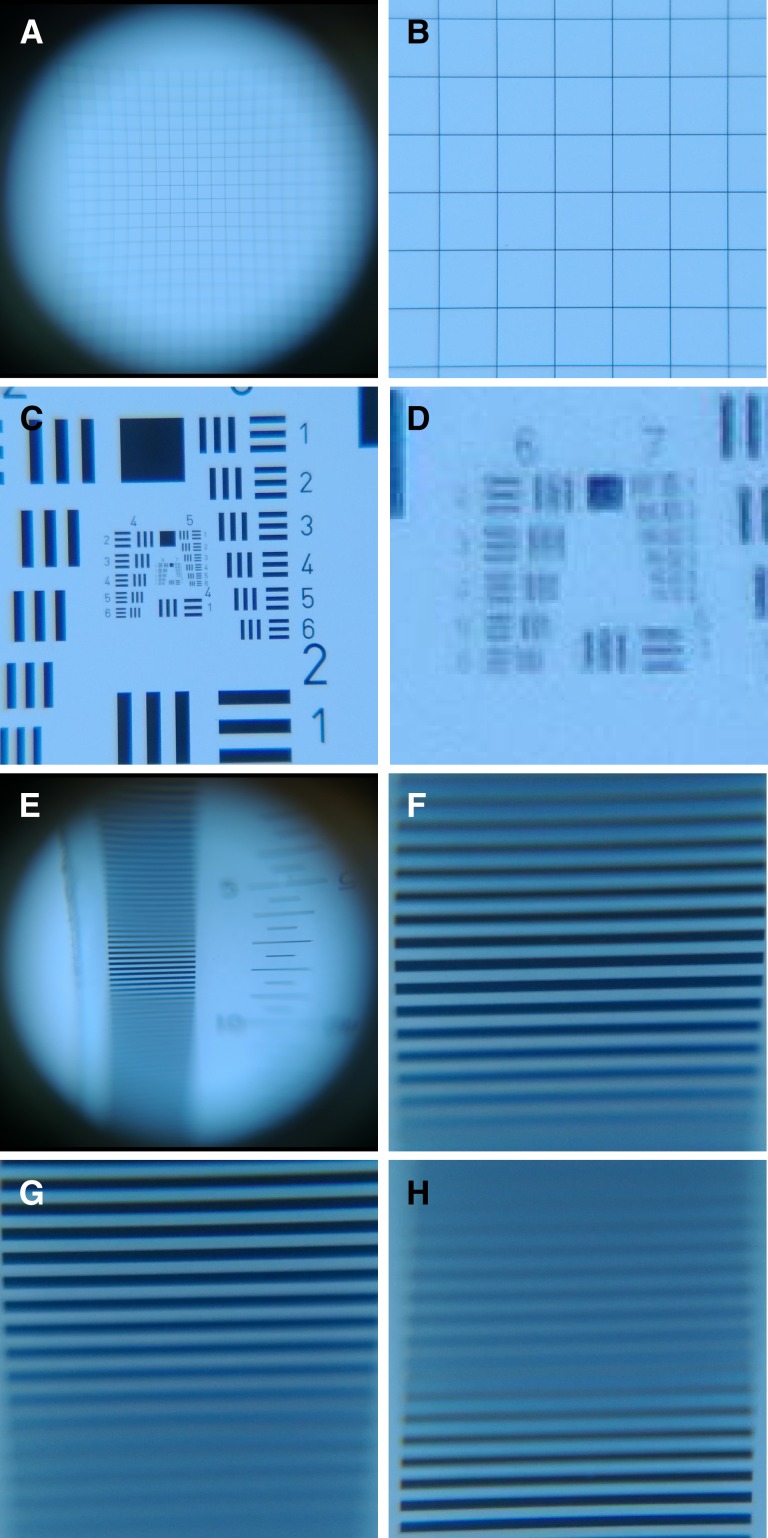
Photo-micrographs of calibration targets. (**A**) 500 μm grid at 1× precapture digital zoom. (**B**) 500 μm grid at 4.5× precapture digital zoom. (**C**) U.S. Air Force (UASF) resolution target at 4.5× precapture digital zoom. (**D**) Postcapture digitally enlarged portion of (**C**) showing elements 6 and 7. (**E**) Depth of field target at 1× precapture digital zoom with the focus set at 500/1,000. (**F**) Depth of field target at 4.5× precapture digital zoom with the focus set at 500/1,000. (**G**) Depth of field target at 4.5× precapture digital zoom with the focus set at 0/1,000. (**H**) Depth of field target at 4.5× precapture digital zoom with the focus set at 1,000/1,000.

**Figure 3.**
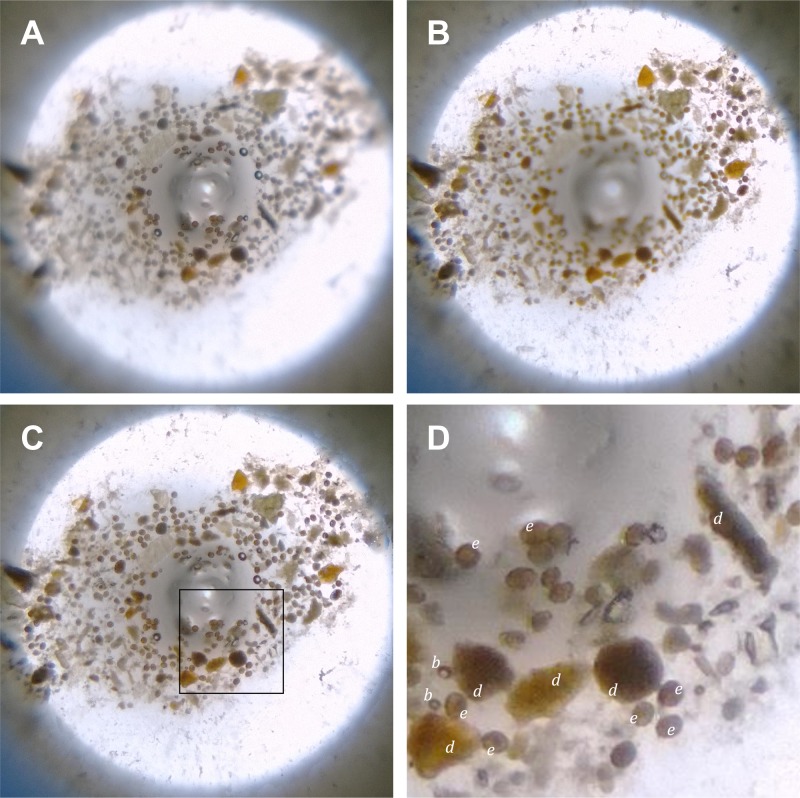
Smartphone microscopy of a slurry of human feces positive for soil-transmitted helminths (STH), that contained buoyant objects accumulated by an axisymmetric meniscus (AxM) formed about the rod (3 mm diameter). (**A**) Image acquired at 4.5× precapture digital zoom with the focus set at 300/1,000. (**B**) Image acquired of the same scene as in (A), except with the focus set at 700/1,000 (**C**) Extended depth of field composite imaged derived from (**A**), (**B**) and a third image of the same scene as in (**A**) and (**B**) except with the focus set at 500/1,000 (not shown). (**D**) Digitally zoomed portion of a region corresponding to the square black frame (inset) in (**C**) indicating example *Ascaris lumbricoides* ova (e), debris (d), and air bubbles (b).

A smartphone photomicroscope image of the grid distortion target taken with no digital zoom with the focus set to the mid-level of the vertical field (500/1,000) was able to capture the complete illuminated field (Figure 2A

). The 24 × 24 squares of the 500 μm grid in the image corresponded to a FOV 12 mm in diameter. Pincushion distortion and defocus due to the field curvature of the objective lens were evident toward the edge of the FOV. Only a central region of 8 × 8 squares (4 mm in diameter) appeared free of distortion. Adjustment of the precapture digital zoom to 4.5× restricted the FOV to 3.5 mm in diameter (Figure 2B). In this configuration, the image of an aligned rod (3 mm diameter) appeared in a single FOV while providing some latitude (0.5 mm) for physical misalignment (Figure 3

). Images of the 1951 USAF resolution target at 4.5× precapture digital zoom (Figure 2C) and digitally enlarged postcapture (Figure 2D), were able to be resolve group 6, element 6 of the optical resolution target, which had 114 line pairs/mm and corresponds to a lateral resolution of 8.8 μm. Images acquired of the depth-of-field target at various focus settings, showed a strip of parallel lines at a frequency of 5 line-pairs/mm (Figure 2E–F). With no digital zoom and the focus set 500/1,000, only the central lines were resolved with sharp focus (Figure 2E). At a precapture digital zoom of 4.5×, with the focus set at 500/1,000 (Figure 2F), 17 lines were contained within the FOV. From this image, five of the centrally located lines were in sharp focus corresponding to a depth of field of 1 mm. Comparison of depth-of-field target images taken at 0/1,000 (Figure 2G) and 1,000/1,000 (Figure 2H) showed a combined depth of focus covering the entire desired vertical range in the FOV, which, for the 17 lines, is 3.4 mm. In this configuration, all of the vertically displaced image features in the AxM were able to be clearly seen. Images of the fecal slurry prepared from the patient positive for *A. lumbricoides* were acquired at various focal depths (Figure 3A and B) and successfully combined into a composite extended depth of field (EDF) image (Figure 3C). In Figure 3A, the central zone, corresponding to approximately a quarter of the diameter of the rod, appears in sharp focus; whereas in Figure 3B, the outer annular zone, corresponding to approximately a quarter of the diameter of the rod appears in sharp focus. The remaining image features are defocussed. The in-focus portions of these two images represent the focal depth boundaries. The EDF image incorporating three equidistant focal slices (Figure 3C) resolved all features of the AxM in sharp focus. Cropped images exhibited no visible pixilation when expanded, and with the rod diameter enlarged to 300 mm (100× digital scaling), *A. lumbricoides* ova are distinguished from other buoyant objects by their color, oval shape, and size (∼60 μm).^9^,^10^

## Discussion

We selected the Nokia Lumia 1020, previously used for photomicroscopy,^29^ because it has a number of desirable features for field-deployable EDF imaging of AxM that include a large (1/1.5” format, 41 megapixel) image sensor with 1.1 μm pixels and adjustable high-performance 6-element Zeiss optics. Images can also be recorded in various image formats. This smartphone is compact (130.4 × 71.4 × 10.4 mm, 158 g), providing a 4.5″ format display (1280 × 768 pixel) and touch screen user interface. Combined with an additional objective lens, this photomicroscopy system provided sufficient resolution for single FOV EDF images of nematode ova accumulated by an AxM. However, the images of *Ascaris* ova lack the internal detail revealed by conventional optical microscopes,^9^,^10^ and would benefit from improved optics yielding higher resolution. Smartphones are constrained as microscopes by their sophisticated optical configurations^23^ and they require dedication to POC applications because of their proximity to potentially infectious material.

Single FOV images of fecal specimens acquired using an AxM and EDF image processing offers significant advantages over traditional STH microscopy by simplifying digital image capture, which enables the transmission of images to centralized experts for interpretation, computer-assisted analysis and the ability to archive and audit image data. Ease of use in the field may also make multiple sampling over time more feasible, mitigating the effects of irregular egg excretion on diagnostic accuracy.^30^ As with other flotation-based STH diagnostics, applications for field-deployable, quality-assured monitoring of *Ascariaisis* and imaging the ova of other helminths such as hookworms, *Trichuris* and schistosomes will require sample preparation and flotation fluid optimization.^16^ Future work will be directed toward species-specific sensitivity assessments and sample processing and imaging improvements.
